# Divided Attention Improves Delayed, but Not Immediate Retrieval of a Consolidated Memory

**DOI:** 10.1371/journal.pone.0091309

**Published:** 2014-03-07

**Authors:** Yoav Kessler, Susan Vandermorris, Nigel Gopie, Alexander Daros, Gordon Winocur, Morris Moscovitch

**Affiliations:** Rotman Research Institute, Baycrest Centre and University of Toronto, Toronto, Ontario, Canada; University of Texas at Dallas, United States of America

## Abstract

A well-documented dissociation between memory encoding and retrieval concerns the role of attention in the two processes. The typical finding is that divided attention (DA) during encoding impairs future memory, but retrieval is relatively robust to attentional manipulations. However, memory research in the past 20 years had demonstrated that retrieval is a memory-changing process, in which the strength and availability of information are modified by various characteristics of the retrieval process. Based on this logic, several studies examined the effects of DA during retrieval (Test 1) on a future memory test (Test 2). These studies yielded inconsistent results. The present study examined the role of memory consolidation in accounting for the after-effect of DA during retrieval. Initial learning required a classification of visual stimuli, and hence involved incidental learning. Test 1 was administered 24 hours after initial learning, and therefore required retrieval of consolidated information. Test 2 was administered either immediately following Test 1 or after a 24-hour delay. Our results show that the effect of DA on Test 2 depended on this delay. DA during Test 1 did not affect performance on Test 2 when it was administered immediately, but improved performance when Test 2 was given 24-hours later. The results are consistent with other findings showing long-term benefits of retrieval difficulty. Implications for theories of reconsolidation in human episodic memory are discussed.

## Introduction

Our memories are dynamic, ever changing and evolving. Our experiences and thoughts are constantly registered into memory, being combined with the vast amount of information that already is there. Encoding new information results in forming new memories and updating old ones, and as a result affects the information that would be retrieved in response to a cue. However, memory change is not limited to encoding. It is now established that retrieval processes play an important role in enhancing or suppressing the *accessibility* of memory representations [1], and in this way affect the probability of retrieving them subsequently. Specifically, the enhancement of retrieval by previous retrieval attempts (testing effect, e.g. [2]), and the suppression of competitor items caused by retrieving an item (retrieval-induced forgetting, [3]) demonstrate that retrieval, in addition to encoding, plays an important role in shaping and changing our memories and the ways we use them.

The finding that retrieval is by itself a memory-changing phenomenon challenges the “standard” distinction between encoding and retrieval processes. Accordingly, previous theoretical and empirical dissociations between these processes need to be reexamined while taking the memory-changing properties of retrieval into account. One such important dissociation concerns the differential effect of divided attention (DA) on encoding vs. retrieval. A large literature demonstrates that encoding is substantially impaired when done under DA, but retrieval is relatively resistant to DA ([4]–[6], but see [7]–[9] for exceptions). This finding led researchers to regard retrieval as relatively automatic and effortless, in contrast to the initial encoding of information which relies more heavily on attentional resources. However, the finding that retrieval is in some cases a memory-changing process implies that some aspects of memory encoding take place during retrieval. If so, then DA during retrieval can be expected to impair performance on a future memory test, even if retrieval per se was not affected by the DA manipulation.

Based on this logic, the effect of DA during retrieval on a subsequent memory test has been examined recently in several laboratories [10]–[12]. In Dudukovic and colleagues’ study [10], for example, a picture appeared in each trial of the study phase, and participants had to answer one of two questions: (a) whether the item is living or non-living, or (b) whether he/she likes or dislikes the presented item. Immediately thereafter, a first recognition test was applied (Test 1), either under full attention (FA) or with a concurrent DA task. A second recognition test (Test 2) was given two days later under FA. The test had the same structure as the first test, and included all the items presented in the study phase, plus new items. Dudukovic et al. found better recognition for items that were tested under FA as compared to DA, demonstrating the “standard” negative effect of DA on future testing. Furthermore, recognition in both the DA and FA conditions was higher than for items that were not tested in Test 1, demonstrating the general advantage of re-testing [2]. Similar results were reported by Guez and Naveh-Benjamin [12], using word stimuli and experimental procedures that minimized the possibility of intentional encoding in Test 1 as an alternative account for the results.

The results described above support the notion of retrieval as an encoding phenomenon by demonstrating that DA during retrieval impairs performance in a subsequent memory test, similar to the hampering effect of DA on encoding. However, a *beneficial* DA after-effect could be expected based on other considerations. Bjork and colleagues [13]–[14] had demonstrated that training procedures that proved to be quick and efficient for learning new skills or materials, resulted in smaller long-term benefit than training procedures in which the study phase was longer and more effortful. In a similar vein, manipulations that present relative difficulty during memory practice, such as spacing (as opposed to massing) and mixing (as opposed to blocking), proved beneficial for long-term delayed retrieval, despite being disadvantageous in the short-term. Based on this logic, Gaspelin and colleagues [11] examined whether DA at retrieval also served as a memory enhancing agent in a subsequent retrieval test. In their study, participants studied English-Swahili word pairs and their memory for them was subsequently tested under FA. Then, a second test employed a FA/DA manipulation. Two days later, a final test under FA was administered. In contrast to the hypothesis regarding the potential long-term advantage of relative retrieval difficulty, and at odds with the findings reported by Dudukovic and collegues [10] and Guez and Naveh-Benjamin [12], the DA manipulation had no effect on the final test performance. The complicated empirical picture described above might result from specific differences in materials and methodology, such as the memory materials (single items vs. paired items) or the amount of practice given before the DA manipulation takes place.

The present study was designed to examine the after-effects of DA during retrieval. The novelty of this work is twofold. First, to the best of our knowledge, all previous research in the field, including earlier work on DA during retrieval, employed a paradigm in which retrieval was tested a few minutes after training, as part of the same experimental session. Importantly, the first several hours after initial encoding are recognized as critical for the formation of stable, long-term memories. This time-limited memory stabilization process is termed *synaptic consolidation* [15]–[16]. Since all previous research examined the effects and after-effects of DA on immediate retrieval, it only addressed retrieval of non-consolidated information, and the role of attention in retrieving consolidated information is, therefore, still unknown.

A second goal of the present work was to examine whether the after-effects of DA at retrieval are modulated by the delay between Test 1 and Test 2. By administering Test 2 immediately after Test 1 or 24-hours later, we examined whether the after-effects observed in Test 2 depend on consolidating the products of retrieval at Test 1.

## Methods

### Ethics

The participants provided their written informed consent to participate in the study. The study was approved by the Research Ethics Board at the University of Toronto.

### Participants

Eighty undergraduate students from the University of Toronto participated in the experiment for partial course credit or monetary compensation. All the participants reported having normal or corrected-to-normal vision, English proficiency, and no psychological or neurological dysfunction or learning disabilities. The participants were randomly assigned to the four experimental groups.

### Materials

160 pictures were taken from Rossion and Pourtois’s database [17], forty for each category, defined as a combination of size (bigger/smaller than a shoebox) and animality (living/non-living). These pictures served as stimuli for the study and test phases of the experiments. These pictures were selected from a total of 260 pictures, based on their fit to each category, as judged independently by three of the authors (YK, SV and AD). Additional pictures served as buffer items, to control for primacy and recency effects. The condition in which each stimulus appeared (target/lure and size/animacy task) was counterbalanced between the participants.

### Procedure

The general procedure for each stage of the experiment will be presented first, followed by a description of the overall design.

#### Study phase

The study phase was divided into two blocks, for the size and animacy tasks respectively. The order of these blocks was counterbalanced between the participants. Each part began with 3 buffer trials to control for primacy. Then, 40 trials were presented, followed by 3 additional buffer trials to control for recency. Accordingly, 80 (study) +12 (buffer) items were presented overall. Each trial began with a 1,000 ms inter-trial interval (ITI). Then, the target item was presented until a response was indicated. The response key mapping remained on the screen throughout the block. A standard QWERT keyboard served for response collection. The keys “p” and “q” served to indicate “big” and “small”, respectively, in the size task, and “non-living” and “living”, respectively, in the animacy task. The subsequent memory tests were not mentioned at this stage, making the learning incidental. The duration of this phase was 5–10 minutes.

#### Test 1

The first testing phase was comprised of a recognition test. The test included 80 items, buffered by 3 items to control for primacy and 3 to control for recency. Among the items, 20 were presented in the study phase with the size task, 20 were presented with the animacy task, and 40 were new. Each trial began with a 2,000 ms ITI. Then, a picture appeared, and the participants had to indicate whether or not it had appeared in the study phase, using one of four responses: yes-sure, yes-unsure, no-unsure, and no-sure. The keys “f”, “g”, “h”, and “j” were used to indicate the responses, respectively. For “yes” answers, a second screen appeared asking to indicate with which task the item was associated using one of four responses: bigger/smaller sure, bigger/smaller unsure, living/nonliving unsure, and living/nonliving sure. The same keys were used to indicate the responses, respectively.

Test 1 was conducted with or without a concurrent DA task, based on that used by Gopie, Craik, and Hasher [18]. Single digits (0–9) were presented auditorily at a 1.5s-rate, and the participants had to repeat aloud every odd digit that was preceded by an odd digit. Hence, the auditory task was continuous and was carried out concurrently with the recognition test. In the FA condition, the participants performed the recognition test without an additional concurrent task.

#### Test 2

The second test phase included 160 (test) +6 (buffer items). The test items were composed of all the 80 studied items (half of them tested in Test 1, and half were untested previously), the 40 lure items from Test 1, and 40 new items. The event sequence within a trial was identical to Test 1, in which the participants were asked whether each item was presented in the study phase or not.

### Design

Testing was done on 3 consecutive days. Table 1 describes the 4 experimental groups.

**Table 1:** Experimental Groups.

Group	Day 1	Day 2	Day 3
1	Study	Test 1 (DA)	Test 2
2	Study	Test 1 (FA)	Test 2
3	Study	Test 1 (DA)+Test 2	
4	Study	Test 1 (FA)+Test 2	

## Results

Tables 2 and 3 provide descriptive statistics for item recognition and source memory by Group and Test. Our analysis focused on hit rate as the dependent measure, allowing us to examine whether items that were correctly recalled in Test 1 were sensitive to our manipulations as observed in Test 2. Parallel analyses on corrected recognition scored showed similar findings as the reported data, and, therefore, were omitted for the sake of brevity (see Table 2). For both item and source analyses “Yes” and “No” responses were collapsed across the “sure” and “unsure” response alternatives, which did not demonstrate any differential effects. Alpha was. 05 in all the analyses.

**Table 2:** Mean (and standard deviation) for recognition performance by Group and Test.

Group	Description		Test 1	Test 2		
				Total	Tested	Untested
1	DA+Delay	% Hits	71.9	59.2	76.4	42.0
			(.12)	(.15)	(.15)	(.18)
		% False-Alarms	23.0	25.3	38.6	12.0
			(.12)	(.14)	(.20)	(.11)
		% Hits minus	48.9	33.9	37.8	30.0
		% False-Alarms	(.12)	(.10)	(.14)	(.15)
2	FA+Delay	% Hits	64.6	48.4	65.6	31.1
			(.15)	(.12)	(.15)	(.13)
		% False-Alarms	12.5	19.5	31.4	7.6
			(.09)	(.10)	(.14)	(.08)
		% Hits minus	52.1	28.9	34.2	23.5
		% False-Alarms	(.16)	(.11)	(.17)	(.09)
3	DA+No-Delay	% Hits	66.6	53.5	66.6	40.4
			(.17)	(.17)	(.16)	(.18)
		% False-Alarms	19.8	19.9	31.5	8.4
			(.10)	(.09)	(.13)	(.07)
		% Hits minus	46.8	33.6	35.1	32.0
		% False-Alarms	(.15)	(.15)	(.17)	(.17)
4	FA+No-Delay	% Hits	69.5	59.8	71.4	48.1
			(.15)	(.16)	(.14)	(.21)
		% False-Alarms	19.4	21.3	32.3	10.3
			(.11)	(.10)	(.16)	(.09)
		% Hits minus	50.1	38.5	39.1	37.8
		% False-Alarms	(.15)	(.14)	(.18)	(.17)

**Table 3:** Proportions of source identification for correctly recognized items by Group and Test.

Group	Description	Test 1	Test 2		
			Total	Tested	Untested
1	DA+Delay	64.4	61.9	60.4	63.8
		(.11)	(.09)	(.11)	(.20)
2	FA+Delay	65.9	63.3	63.3	63.0
		(.11)	(.10)	(.11)	(.12)
3	DA+No-Delay	57.2	63.0	62.4	63.6
		(.09)	(.09)	(.10)	(.14)
4	FA+No-Delay	62.7	64.7	63.5	67.0
		(.08)	(.06)	(.07)	(.13)

### Performance in Test 1

Our first analysis examined whether DA during Test 1 affected performance in this test. Based on the reviewed literature, a null effect was expected. For this analysis, performance in a pooled FA group (composed of Groups 1 and 3) was compared to a pooled DA group (composed of Groups 2 and 4). As expected, the DA manipulation did not affect Test 1. For item memory, hit rates were 67.1% and 69.3% for the FA and DA groups, respectively, *F(1,78) = .44, MSe = .0220, η_p_^2^ = .01, p = .51*. Source memory, defined as the conditional probability of recalling the correct task at encoding in items that were classified correctly as “old”, was 64.3% and 60.8% for the FA and DA groups, respectively, *F(1,78) = 2.28, MSe = .0105, η_p_^2^ = .03, p = 13*. The absence of DA effect on Test 1 therefore sets the stage for the subsequent analyses.

### Main Analysis

The main analysis focused on performance at Test 2. A three-way ANOVA was conducted on hit rates in Test 2 with Attention (FA vs. DA) and Delay (short vs. long test phase) as between-subject variables, and Testing (tested vs. untested items at retrieval) as a within-subject variable. The main effect of Testing was significant, *F(1,76) = 411.95, MSe = .0085, η_p_^2^ = .84, p<.000001*, reflecting higher hit rates for tested (70%) as compared to untested items (40.4%). The two-way interaction between Testing and Delay was significant, *F(1,76) = 11.04, MSe = .0085, η_p_^2^ = .13, p<.005*. This interaction stems from a marginally-significant effect of Delay for untested items, 44.3% vs. 36.6% for the short and long delays, respectively, *F(1,76) = 3.68, MSe = .0321, η_p_^2^ = .05, p = .06*, but no effect for tested items, 69.0% vs. 71.0% for the short and long delays, respectively, *F(1,76) = .35, MSe = .0226, η_p_^2^ = .004, p = .55*. Finally, the 2-way interaction between Attention and Delay was significant, *F(1,76) = 6.30, MSe = .0462, η_p_^2^ = .08, p<.05*. No other effects were significant (all Fs<1).

**Figure 1:**
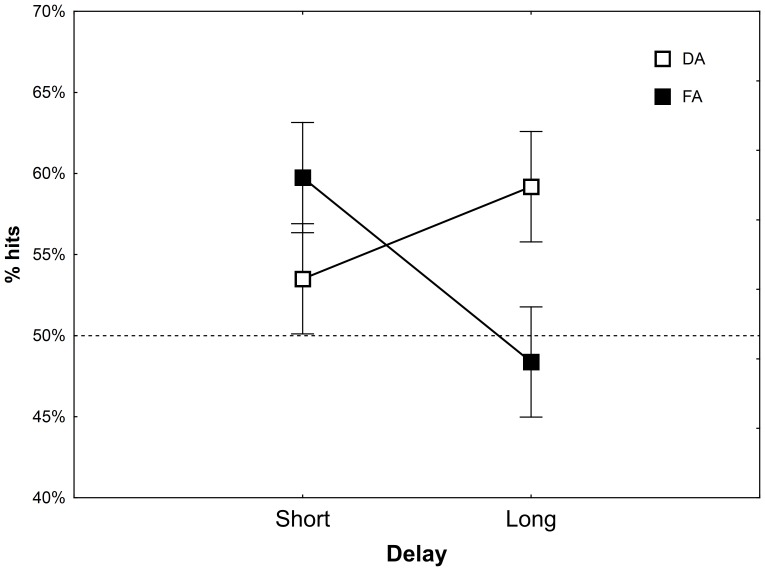
Hit rates in Test 2 as a function of Attention and Delay. Error bars represent the standard error of the mean. Hit rates did not differ between the attention conditions in the short delay, but were higher for the DA group in the long delay, in contrast to the prediction of the reconsolidation hypothesis. The dashed line represent chance performance (hits = 50%).

We turned to explore the interaction between Attention and Delay (see Figure 1). The results show an opposite pattern of DA effects on the Test phase performance, in the different delay periods. The attention manipulation at retrieval did not affect Test 2 after a short delay significantly: hit rates were 59.8% and 53.5% for the FA and DA groups, respectively, *F(1,76) = 1.69, MSe = .0231, η_p_^2^ = .02, p = .20*. In contrast, performance after a long delay was better for the DA group compared to FA, 59.2% and 48.4%, respectively, *F(1,76) = 5.06, MSe = .0231, η_p_^2^ = .06, p<.05*.

### Source Memory

The 3-way ANOVA on source memory for the task at study did not demonstrate any main effect or interaction, all Fs<1. Notably, this was not due to a diminished memory for the source. The proportion of correctly remembered items for which source memory was correct was higher than 60% in all groups, both for tested and untested items, and significantly higher than 50%, t(19)>28.81, d>6.45 in all groups.

### Memory for Items that were Correctly Recognized in Test 1

The previous analysis examined overall performance in Test 2, regardless of whether the recalled items were previously recalled in Test 1. A more refined analysis is required in order to test the fate of items that were correctly identified as old in Test 1. This is especially important since these are the items that were encoded in the study phase and were actually retrieved in Test 1. We ran an ANOVA on Test 2 data of Groups 1–4 with Attention (FA vs. DA) and Delay (short vs. long) as between-subject variables. The dependent variable was conditional hit rate in Test 2 for items that were correctly recognized as old in Test 1. Only the 2-way interaction was significant, *F(1,76) = 4.32, MSe = .0169, η_p_^2^ = .05, p<.05*. The data mirrored the findings of the main analysis described above. Performance was better for FA compared to DA in the short delay, 87.8% vs. 80.4%, respectively. The opposite pattern was observed in the long delay, 79.4% vs. 84.0% for FA vs. DA, respectively. Accordingly, this analysis supported the general findings observed in the main analysis. A similar ANOVA was conducted on the source memory data. None of the effects was significant, all *F’s<1*.

## Discussion

The goal of the present study was to examine the effects of divided attention during memory retrieval on a subsequent memory test. The divided attention manipulation was carried out while testing on consolidated memory items, which were studied 24 hours before. Our findings can be summarized as follows. First, the standard finding that DA during retrieval does not impair performance was replicated in the data of Test 1. This is important, since comparing performance in Test 2 depends on the assumption that performance was equal among the groups at baseline. Second, the negative effect of DA during Test 1 on an immediate Test 2 is weak or even absent. In contrast, a beneficial effect is observed when Test 2 is delayed. This key finding is in line with our above predictions based on Bjork’s work [19].

The null effects on source memory performance in our study warrant an explanation. Although source memory for correctly recalled items was above chance for all conditions, performance did not interact with either Attention or Delay. We interpret the results as stemming from a floor effect. Unlike item recognition, source memory depends heavily on recollection. By the time Test 1 was given, this process might already be diminished to such a degree that it was no longer sensitive to our manipulation.

Our results extend the findings of Dudukovic et al. [10]. While Dudukovic et al. found that DA during testing hampered performance in a later delayed test, we found the opposite pattern. The major difference between the experimental procedures is the delay between the study phase and Test 1. Dudukovic et al. administered Test 1 immediately after the study phase, and therefore the test (and the DA manipulation) likely targeted labile, pre-consolidated material. Testing within a few hours from the study phase, therefore, can be regarded as an instance of trace alteration. By contrast, having a 24-hour delay period in our study ensured that Test 1 was carried out on consolidated material. Taken together, the results of the two studies show that the mechanisms by which testing enables re-learning are different, depending on the nature of the tested representations. Re-learning requires attention when carried out immediately after study, but is rather immune to DA (and perhaps benefits from it) when done outside the consolidation window.

In line with our predictions, the paradoxical effect of attention in our study parallels previous findings on the relationship between practice difficulty and subsequent test performance [19]. Specifically, re-learning items during Test 1 under DA resulted in better performance in a later test, but only when the test was delayed. One mechanism that can account for this long-term benefit observed in our study is contextual variability. Since the learning and re-learning contexts differed more in the DA condition compared to FA, more distinctive retrieval cues were created for the items in the DA condition. The benefit of retrieval cues variability is only observed in a delayed test. In this case, the strength of the study phase and Test 1 contexts is more equated compared to our short delay condition, in which the context of Test 1 was stronger due to recency.

In a broader context, our results shed light on the limits of reconsolidation in human episodic memory. Specifically, the standard model of synaptic consolidation suggests memories are stable and persistent after consolidation is completed, and there is no further loss of information. In contrast, the reconsolidation hypothesis argues that consolidation is not only needed for newly encoded memories, but also after retrieving old memories. Accordingly, long-term memories become labile and unstable once retrieved, and hence need to be reconsolidated again in order to be retained. Critically, when reconsolidation is prevented, the original representation will be lost. While initial work on reconsolidation focused on fear learning in rodents (e.g., [20]), several groups have recently argued for a reconsolidation process in human episodic memory, using behavioral manipulations as amnestic agents [21]–[26].

Since our design involved testing and re-testing of consolidated materials, it can be asked whether or not Test 1 triggered reconsolidation, and whether this process was affected by the DA manipulation. In fact, the question, whether every act of retrieval destabilizes memory and therefore triggers reconsolidation, is still open. While earlier work suggested that reconsolidation might always be triggered following retrieval ([27]; see [28] for review), more recent studies suggested that reconsolidation only occurs following a mismatch between the new and old information [29]–[30].

Our results support the latter view. If retrieval in Test 1 triggered reconsolidation, then our DA manipulation that typically impairs encoding, would be expected to impair this process. Hence, a negative after-effect of DA would have been observed outside the reconsolidation window, namely in the long delay condition. However, our results are the opposite to this prediction- DA at retrieval *benefited*, rather than impaired, performance in a subsequent memory test. Since our paradigm did not include separate reminder and interference stages, it is difficult to know whether or not retrieval in Test 1 triggered a process which made the original memory traces labile. But, even if they did, the DA manipulation did not impair the subsequent reconsolidation of the information. In either case, our results are inconsistent with the reconsolidation hypothesis.

### Conclusion

Our study is the first to demonstrate the beneficial after-effect of DA during retrieval, on a future memory test. Critically, as predicted by Bjork and colleagues [19], this benefit is only observed when ample time is given between the two tests. Moreover, the long-term benefit is not limited to the tested items but extends to the entire list. An important finding is that consolidation after the initial study and before Test 1 is an important precondition for our results. Our finding of a delayed DA benefit is at odds with the results of Gaspelin and colleagues [11], who did not find any effect of DA on a subsequent test. As suggested above, a key difference between the studies is the insertion of a 24-hour delay between the study phase and Test 1 in the current study. Theories of episodic memory reconsolidation are unable to account for our data. Future empirical and theoretical work is needed to establish the role of consolidation and re-consolidation in the processes that underlie the after-effects of memory retrieval.
